# Valorization of agro-forest wastes (oak acorns, vineyard pruning, and olive pruning) through the cultivation of shiitake (*Lentinula edodes*) mushrooms

**DOI:** 10.1016/j.heliyon.2024.e32562

**Published:** 2024-06-15

**Authors:** Youssef Najib Sassine, Stephanie Nabhan, Elina Rachkidy, Zeina El Sebaaly

**Affiliations:** a Lebanese University, Faculty of Agriculture, Department of Plant Production, Beirut, Lebanon; b University of Forestry, Faculty of Agronomy, Department of Agronomy, Sofia, Bulgaria

**Keywords:** Mushrooms, Lignocellulose, Alternative substrates, Recycling, Nutritional value

## Abstract

Experimental research has been focusing on developing new substrates for growing shiitake mushrooms as alternatives to the standard oak sawdust substrate. The selection of appropriate lignocellulosic materials is based on their availability in the production area and their compatibility with the requirements of the mushroom species being cultivated. In comparison to oak sawdust substrate (OS) as the control, this study evaluated the potential of oak acorns (OA), olive pruning (OLPR), and vineyard pruning (VIP), and various combinations: OA-OLPR:1-1, OA-VIP:1-1, OS-OLPR:1-1, and OS-VIP:1-1, prepared on a dry weight basis. In comparison to OS, complete mycelial development was hastened in OA, OA-VIP: 1-1, and OS-VIP:1-1 by 9.5, 7.9, and 4.2 days and delayed in OLPR and OS-OLPR:1-1 by 11.3 and 7.0 days, respectively. Also, harvest was earlier in OA, OA-VIP:1-1, and OS-VIP:1-1 by 9.3, 6.7, and 3.3 days, respectively, while it was significantly delayed in OLPR, VIP, and OS-OLPR:1-1 by 12.3, 3.7, and 8.0 days, respectively. While the total biological yield was significantly reduced in OLPR, OS-OLPR:1-1, VIP, and OS-VIP:1-1, it was comparable to OS in OA, OA-OLPR:1-1, and OA-VIP:1-1 (597.0, 552.0, 532.2, and 556.2 g/kg, respectively). Production was consistently high over two consecutive flushes in OS, OA, and OA-VIP: 1-1. Total biological yields were higher in OA-OLPR: 1-1 than OS-OLPR:1-1 and in OA-VIP:1-1 than OS-VIP:1-1. OA increased mushroom number and firmness, VIP and OLPR increased mushroom weight, and OA-VIP:1-1 increased pileus thickness. Mushrooms’ protein and fiber contents were higher than OS in all substrates and the highest in OA-OLPR:1-1 (8.7 %) and OLPR (2.8 %), respectively. Conclusively, the substrates OA, OA-VIP: 1-1, and OA-OLPR:1–1 may alternate oak sawdust; however, the first two substrates have an advantage over the third due to earlier harvests. Also, it is more favorable to use VIP and OLPR in combination with OA than to use them alone.

## Highlights

• Oak acorns alone or mixed in equal proportions with olive pruning or vineyard pruning may alternate with oak sawdust.
• Oak acorns increase the mushroom number, while olive and vineyard pruning increase mushroom weight.
• The use of oak acorns alone or mixed with vineyard pruning caused an earlier harvest than oak sawdust.
• Mushrooms' protein and fiber contents were ameliorated on agro-forest wastes compared to oak sawdust.

## 1 Introduction

The Japanese mushroom Shiitake (*Lentinula edodes*) is favored for its enticing aroma and unique flavor. It has gained popularity because of its numerous health benefits such as its anti-carcinogenic, anti-HIV, and anti-microbial effects [1,2] and its high nutritional value and high levels of vitamins (B1, B2, and B3) and minerals like calcium, magnesium, and iron [3]. Globally, it is the second most cultivated mushroom next to the button mushroom (*Agaricus bisporus*), accounting for around 22 % of the global mushroom production [4,5].

In the wild, shiitake is found growing on oak or similar hardwood trees of the Fagaceae family [6]. On an industrial scale, the conventional substrate used is composed of 80 % hardwood sawdust (mainly oak sawdust) and 20 % starch-rich additives such as wheat bran or rice bran [7]. In many countries, the use of oak sawdust is a limiting factor and poses an environmental risk because of the need to cut oak trees [8]. In general, mushroom farmers use lignocellulosic materials that are easily available in their area and meet the needs of the species of mushrooms they are cultivating [9]. Mushrooms degrade the lignocellulosic materials of the growing substrate through their ligninolytic enzymatic complex and utilize the degraded products to produce their fruiting bodies [10,11]. Experimental research has shown that shiitake may be grown using a variety of agro-industrial wastes, including sugarcane bagasse [12], corncobs [13], sunflower seed hulls [14], vineyard pruning [15], etc.

In Lebanon, viticulture ranks third among fruit crop production [16], and olive tree cultivation covers 23 % of the total agricultural land [17]. Therefore, large amounts of waste are produced because of the pruning activities at vineyards [18] and olive orchards [19]. Most of the olive pruning (OLPR) and vineyard pruning (VIP) is improperly handled; it is either burned or disposed of on the sides of the orchards. According to early studies, both types of waste have potential for mushroom cultivation. For instance, VIP hastened the mycelial growth of shiitake mushrooms [20] and improved the biochemical composition [21], minerals, and protein contents [22] of mushrooms. Additionally, the use of OLPR in proportions of 17, 66, or 75 % (proportions of volume) improved the protein, crude fiber, iron, and mono-unsaturated fatty acid contents of oyster mushrooms (*Pleurotus ostreatus*), and its application in 25 % accelerated mycelia run, fruit formation, and harvest [19,23]. Furthermore, around 52.4 % of Lebanon's total forest area is covered by oak forests [24,25]. Large quantities of oak acorns (OA) are therefore generated. Li et al. [26], Taib et al. [27], Rababah et al. [28], and Vinha et al. [29], all asserted that oak acorns are high in fatty acids, minerals, and proteins, and their starch content is 55 %, making them richer than wheat bran, the most common supplement used for shiitake cultivation. Similarly to OLPR, oak acorns have the potential to be used for the cultivation of shiitake; however, the use of both waste types has not yet been investigated.

Therefore, this study is the first trial that investigates the prospects of using locally available VIP, OLPR, and OA, either separately or in various combinations, to grow shiitake strain 3782. Furthermore, it looks into alternative local substrate formulations for shiitake production in addition to the standard substrate made of oak sawdust.

## 2 Materials and methods

### 2.1 Tested substrates

The experiment, was carried out at the Research and Training Center, Ghazir station, Lebanon. It assessed eight treatments (substrate formulations) that were set up in a completely randomized design and replicated six times (six bags of 1 kg each). Tested substrate formulas were assigned as: OS (oak sawdust), OA (oak acorns), VIP (vineyard pruning), OLPR (olive pruning), OA-OLPR:1-1, OA-VIP:1-1, OS-OLPR:1-1, and OS-VIP:1-1, where OS was used as a control. Also, 200 g of wheat bran was added equally to all substrates, which were prepared on a dry weight basis. Olive pruning and vineyard pruning were ensured from local organic orchards situated in the Bekaa region, and oak acorns were collected from a local oak forest situated in Jbeil, Lebanon. A Pro Woodchipper was used to reduce the size of olive wood, vine wood, and oak acorns to sawdust. Oak sawdust was sourced from ‘Mushies’, a local mushroom grower, and oak wood logs were initially collected from local forests.

### 2.2 Analysis of substrate properties

Analytical tests were performed to assess the physico-chemical properties of each substrate (Table 1) at the Lebanese Agricultural Research Institute (LARI)-Tal Amara station using a sample of 0.5 kg.

**Table 1:** Properties of substrates used to grow shiitake.

	OS	OA	OLPR	VIP	OA-OLPR	OA-VIP	OS-OLPR	OS-VIP
Dry matter (%)	91.3	86.8	93.0	92.6	90.7	92.9	92.8	93.4
Moisture (%)	8.7	13.2	7.0	7.4	9.3	7.1	7.2	6.6
C (%)	53.80	56.43	50.89	56.35	54.13	55.01	54.71	55.24
N (%)	0.49	0.58	1.07	0.75	1.02	0.68	0.62	0.84
C/N ratio	109.8	97.3	47.6	75.1	53.10	80.89	88.2	65.76
Organic matter	92.5	97.1	87.5	96.9	93.1	94.6	94.1	95.0
Crude protein	3.0	3.6	6.7	4.7	6.4	4.3	3.9	5.3
Ash content	7.45	2.94	12.46	3.1	6.90	5.4	5.89	5.0
Fat (%)	0.7	1.0	2.1	0.8	2.5	3.6	3.9	4.7
Carbohydrates	80.0	79.2	71.7	84.0	74.9	79.7	79.1	78.5
NDF (%)	77.2	61.3	57.8	82.6	59.2	75.0	73.6	77.8
ADF (%)	68.0	44.0	45.7	60.6	48.0	61.1	59.1	68.7
ADL = lignin (%)	29.2	21.4	22.0	26.7	22.1	35.3	29.6	29.9
Cellulose (%)	38.8	22.6	23.7	33.9	26.1	25.8	29.4	38.8
Hemicellulose (%)	9.1	17.2	12.1	22.0	11.2	13.9	14.5	9.1
pH	5.4	5.4	7.2	5.9	6.2	5.6	6.8	5.5

NDF: neutral detergent fiber, ADF: Acid detergent fiber, ADL: Acid detergent lignin, OS: Oak sawdust, OA: Oak corns, OLPR: olive pruning, VIP: Vineyard pruning.

The pH of substrates was evaluated on the filtrates of substrate samples using a pH-meter (ADWA 132 CE). Moisture content was determined following AOAC [30] by drying 2 g of substrate samples under pressure of 100 mm Hg to a constant weight at 95–100 °C. The dry matter content was then calculated as given in Equation (1):

(1) Drymatter(%)=100−moisture(%)

Ash content was determined according to AOAC [30]: 2 g of sample were weighed into a porcelain crucible and preheated to 600 °C for 2 h in a temperature-controlled furnace. Then, the crucible was transferred to a desiccator, cooled, and weighed, and the percentage of ash was calculated as given in Equation (2):

(2) $$Ash\left(\%\right)=\frac{\;\text{Initial}\;\text{weight}\;\text{of}\;\text{sample}\;\left(W1\right)\left(g\right)–\;\text{weight}\;\text{of}\;\text{sample}\;\text{after}\;\text{ash}\;\text{drying}\;\left(W2\right)\left(g\right)}{\text{Initial}\;\text{weight}\;\text{of}\;\text{sample}\;\left(W1\right)\;\left(g\right)}$$

Crude fat was determined by acid hydrolysis according to AOAC [31], and crude protein was assessed using the Micro-Kjeldahl method (N × 6.25) [32]. Organic matter, carbon, nitrogen, and carbohydrate contents were calculated as given in the Equations ((3), (4), (5), (6)):

(3) Organicmatter(%)=100−ash(%)

(4) $$\text{Carbon}\;\left(\%\right)=\frac{\text{Organic}\;\text{matter}\;\left(\%\right)}{1.72}$$

(5) Nitrogen(%)=Crudeprotein(%)x6.25

(6) Carbohydrates(%)=100−moisture(%)−crudeprotein(%)−crudefat(%)−minerals(%)

Crude fibers were assessed by the Weende technique according to AOAC [33]. Fiber fractions (acid detergent fiber: ADF, neutral detergent fiber: NDF, and acid detergent lignin: ADL) were measured by the Fibertherm methodology according to Wannasawang et al. [34]. Therefore, cellulose, hemicellulose, and lignin contents were calculated as given in Equations ((7), (8), (9)):

(7) Lignin(%)=ADF(%)

(8) Hemicellulose(%)=NDF(%)−ADF(%)

(9) Cellulose(%)=ADF(%)−ADL(%)

### 2.3 Substrate preparation and inoculation

Initial sawdust materials of the different substrate formulations (OA, OS, OLPR, and VIP) underwent a first pasteurization for 15 min in boiling water at 100^o^C to remove any dirt, insects, or any fungal or bacterial contamination. Then they were left to drain and sun-dry for several days to get rid of excessive moisture. Wheat bran was added to the different sawdusts used alone or in combination, and the resulting mixtures were wetted to a moisture level of 60–70 % [35]. Wetted substrates were then filled into autoclavable polypropylene filter patch bags of 1 kg each, sealed, and steam pasteurized in modified barrel steamers for 6 h at 120 °C and 100 mbar. Afterwards, autoclaved bags were cooled down in a room set at 16 °C until the substrate temperature decreased below 25 °C for an appropriate spawning process and suitable mycelium growth [36]. Grain spawn of shiitake (strain 3782) was applied at a rate of 2 % at the top of the substrate in each bag under hygienic conditions. Then, the bags were labelled according to the corresponding substrate formula.

### 2.4 Incubation and fruit induction

Inoculated substrates were incubated in complete darkness at a temperature of 20–22 °C and a relative humidity of 50–60 %. Temperature and relative humidity inside the incubation room were monitored and kept constant using the humidity/temperature meter (Lutron HT-3007SD). During incubation, five consecutive stages of mycelial development were distinguished based on Chen [37], as follows.

- Stage 1: mycelia run: thin layer of white hyphae completely covering the block.
- Stage 2: mycelia coat formation: hardening of the mycelia sheet covering the whole substrate surface.
- Stage 3: bump formation: clumps of mycelia developing into popcorn shape.
- Stage 4: browning/pigmentation: mycelia block developing a dark brown and dry outer protective layer.
- Stage 5: coat hardening: hardening of the dark coat.

**Fig. 1:**
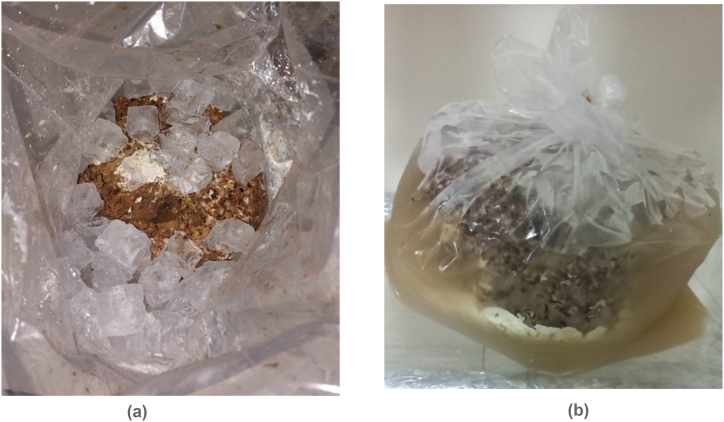
a Soaking procedure; ice cubes added to plastic bags containing substrate blocks of shiitake. bClosed bags containing soaked blocks of shiitake.

The end of each stage was recorded as the number of days after spawning (DAS). At the end of stage 5, and to induce fruiting, polyethylene bags were removed, and the blocks were subjected to a thermal shock by soaking them in bags containing ice cubes for 24 h (Fig. 1 a,b) [38]. Twenty-four hours later, blocks were removed from the soaking bags and put on shelves inside the fruiting room set at a temperature of 16^o^C, a relative humidity of 90 %, and where artificial light of 2000 lux was provided. Inside this room, high relative humidity was maintained using the humidifiers (SANI-JET AIR 2836/P0). After three to five days, pinheads started to appear, marking the beginning of the fruit formation stage, and the number of days required for the appearance of distinct pinheads on blocks was recorded in DAS. At this stage, the blocks were watered daily, three times a day, until the full development of the mushrooms. Depending on the substrate, harvesting took place four to five days after pinhead initiation; when the mature mushrooms showed opened caps with slightly curved outer edges, and apparent gills [39]. The number of days to harvest was also recorded in DAS. Mushrooms were harvested over two consecutive flushes (flush 1: F1 and flush 2: F2). At the end of the first harvest or flush, the blocks were incubated again in darkness at a temperature of 20–22 °C and a relative humidity of 60 %. Afterwards, blocks were soaked again in ice for 24 h to induce a second flush (F2).

### 2.5 Evaluation of production

At each flush, the mushroom number and the mushroom weight were recorded per bag. Also, at both flushes, the biological yield was evaluated as the total weight of mushrooms harvested per bag (BYF1 and BYF2). The total biological yield TBY was calculated by the summation of BYF1 and BYF2 for each treatment, and the biological efficiency was calculated for each treatment according to Yang et al. [40], as given in Equation (10):

(10) $$\text{BE}\;\left(\%\right)=\frac{\text{total}\;\text{biological}\;\text{yield}\;\left(g/\text{bag}\right)}{\text{initial}\;\text{dry}\;\text{weight}\;\text{of}\;\text{substrate}\;\left(g\right)}\;x\;100$$

At the first flush, pileus diameter (PD), pileus thickness (PT), stipe diameter (SD), and stipe length (SL) were assessed on harvested mushrooms using a sliding caliper, and the ratio PD/SL was calculated. Mushroom firmness was measured on five different points of the pileus using a setamatic penetrometer (Stanhope-Seta), and the average value was calculated for each sample.

### 2.6 Evaluation of mushroom composition

Fresh, representative samples of each treatment were used to evaluate their nutritional composition. Analytical tests on mushrooms were performed in triplicates. The moisture content was tested using a moisture analyzer (M5-Thermo A64 M). The ash content was determined by macerating 5 g of mushrooms, putting them in crucible cups, and heating them in a muffle furnace (Carbolite Furnace OAF 10/1) at 550^o^C for 24 h. The ash content was calculated as in Equation (11):

(11) Ash(g)=W2(g)−W1(g)

W2: weight of the crucible containing ash.

W1: weight of an empty crucible.

Fat content was assessed using the Soxhlet apparatus technique [41] by weighing 10 g of mushrooms, macerating them with sea sand, and placing them in the extractor. The round bottom flask of the extractor was weighed (W1) and filled with 500 mL of hexane. The system was heated, and hexane was evaporating, extracting fat, which remained at the bottom of the flask. This cycle was repeated several times for 12 h. Then, the bottom flask containing fat and hexane was put in a bain-marie, hexane was evaporated, and fat remained in the bottom flask that was weighed (W2). Fat content was calculated and then expressed in percentage, as in Equation (12):

(12) Fat(g)=W2(g)−W1(g)

W1: Weight of the empty bottom flask.

W2: Weight of the bottom flask containing fat.

Crude fiber content (%) was determined according to AOAC [42] as the loss of ignition of dried residue remaining after digesting the mushroom sample in 1.25 % (w/v) H2HSO4 and 1.25 % (w/v) NaOH.

Crude protein content (N x 4.38) (%) was determined using the macro-Kjeldhall method based on Reis et al. [43]. Carbohydrates content was then calculated by using Equation (13):

(13) Carbohydrates(%)=100−(crudefiber(%)+crudeprotein(%)+fat(%)+moisture(%)

Ascorbic acid (vitamin C) (mg/100g) was assessed by the titration method using 2.6 Dichlorophenolindophenol [44] by grinding a known weight of mushroom sample, adding 5 % metaphosphoric acid solution and stirring the mixture for 30 min. Then, the mixture was filtered using a suction pump and a Whatman No. 42 filter paper. Ten milliliters of the filtrate were pipetted into a 250 mL conical flask and titrated with 0.025 % of 2.6 Dichlorophenolindophenol reagents. The amount of vitamin C in each extract was calculated using Equation (14):

(14) $$mg\;of\;ascorbic\;acid\;per\;100\;g=\frac{AxIxV1x100}{V2xW}$$

A = quantity of ascorbic acid (mg) reacting with 1 mL of 2.6 indophenol;

I = volume of indophenol (in mL) required for the completion for the titration with extract;

V1 = total volume of extract;

V2 = volume of extract used for each titration.

W = weight of the mushroom sample extracted.

Vitamin D_2_ as ergocalciferol and vitamin D_3_ as cholecalciferol (in μg/100 g) were assessed by high performance liquid chromatography (HPLC) based Mattila et al. [45].

### 2.7 Statistical analysis

Data analysis applied the one-way ANOVA, and means were compared by Duncan's multiple range test at *P*_*value*_<0.05 using SPSS25 version 26. An independent sample *t*-test compared production between Flushes 1 and 2. Also, a stepwise regression was applied to evaluate the relationship between the total biological yield (dependent variable) and productive indicators (independent variables).

## 3 Results and discussion

### 3.1 Mycelia run, fruit formation, and harvest

**Fig. 2:**
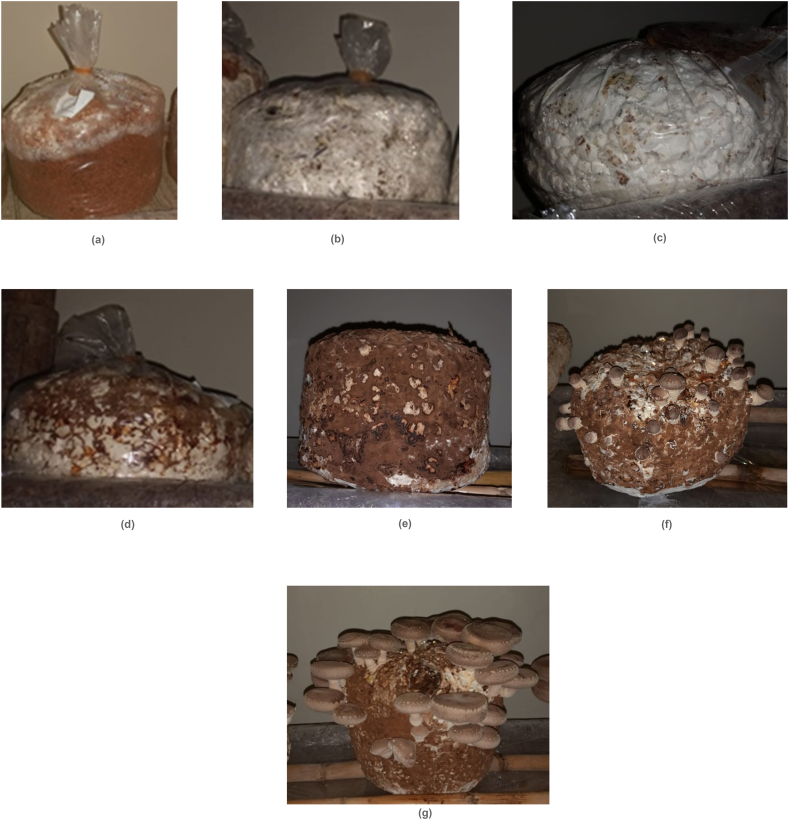
a Substrate blocks at stage 1: mycelial run. b Substrate blocks at stage 2: coat formation. c Substrate blocks at stage 3: bump formation. d Substrate blocks at stage 4: pigmentation. e Substrate blocks at stage 5: coat hardening. f Substrate blocks at pin head formation stage. g Substrate blocks at fruit development stage.

According to the results of one-way ANOVA (Table 2), there was no significant difference in the average number of days required to complete stages 1 and 2 of mycelia growth on tested substrates compared to control, except for a significant delay in stage 2 on OLPR (by 4.6 days). On the other hand, the average number of days to reach stages 3, 4, and 5 was significantly reduced compared to control in OA (by 3.6, 7.5, and 9.5 days, respectively) and in the mixture OA-VIP:1-1 (by 3.6, 6.3, and 7.9 days, respectively). Further, stages 4 and 5 were also hastened in the mixture OS-VIP:1-1 compared to the control; by 3.6 and 4.2 days, respectively. Further, compared to OS, all stages of mycelial growth were significantly delayed in OLPR, with a delay of 11.3 days in stage 5. Also, the last stages of mycelial development (stages 4 and 5) were significantly delayed on the substrate OS-OLPR:1-1 (by 5.9 and 7.0 days, respectively). Findings also showed that the number of days to fruit formation and harvest was significantly lower than OS in OA, OA-VIP:1-1, and OS-VIP:1-1 (reduction by 9.7, 7.5, and 4.2 and 9.3, 6.7, and 3.3 days, respectively). On the contrary, fruit formation was delayed by around 11.9, 3.2, and 7.2 days and harvest by around 12.3, 3.7, and 8.0 days, in OLPR, VIP, and OS-OLPR:1-1, respectively, compared to control. Both stages were reached in a comparable number of days in OA-OLR:1-1 and control. Consecutive stages of mycelia run, pinhead, and fruit formation are shown in Fig. 2 a,b,c,d,e,f,g.

**Table 2:** Number of days (means ± SD) to the different stages of mycelia development, fruit formation, and harvest recorded on tested substrates.

Substrates	Stage 1 (DAS)	Stage 2 (DAS)	Stage 3 (DAS)	Stage 4 (DAS)	Stage 5 (DAS)	Fruit formation (DAS)	Harvest (DAS)
**OS (control)**	15.7 ± 1.0 ab	22.8 ± 1.9BCE	37.3 ± 2.7 b	61.3 ± 3.1c	72.2 ± 2.1c	75.5 ± 1.4 d	79.5 ± 1.5 d
**OA**	12.7 ± 2.3 b	20.7 ± 2.6BCE	33.7 ± 2.3c	53.8 ± 4.5e	62.7 ± 4.8e	65.8 ± 4.3f	70.2 ± 4.4g
**OLPR**	17.4 ± 5.2a	27.4 ± 5.1a	43.8 ± 5.5a	71.8 ± 5.0a	83.5 ± 3.7a	87.4 ± 3.5a	91.8 ± 3.4a
**VIP**	14.3 ± 1.2 ab	22.2 ± 1.0BCE	37.7 ± 1.6 b	64.3 ± 1.4BCE	74.8 ± 0.8c	78.7 ± 1.0c	83.2 ± 1.5c
**OA-OLPR:1**–**1**	13.5 ± 2.6 b	23.7 ± 3.5BCE	38.2 ± 1.9 b	63.2 ± 2.4c	74.0 ± 1.9c	77.5 ± 1.5cd	81.8 ± 1.2cd
**OA-VIP:1**–**1**	13.3 ± 2.0 b	20.8 ± 1.9BCE	33.7 ± 1.6c	55.0 ± 1.8de	64.3 ± 1.8e	68.0 ± 1.3f	72.8 ± 1.0f
**OS-OLPR:1**–**1**	14.5 ± 2.7 ab	24.0 ± 3.2 b	38.2 ± 2.2 b	67.2 ± 2.3 b	79.2 ± 1.2 b	82.7 ± 1.2 b	87.5 ± 1.0 b
**OS-VIP:1**–**1**	12.7 ± 1.8 b	20.0 ± 1.8c	34.8 ± 1.6BCE	57.7 ± 1.9 d	68.0 ± 1.9 d	71.3 ± 1.8e	76.2 ± 1.2e
*P value*	0.048	0.002*	0.000*	0.000*	0.000*	0.000*	0.000*

DAS: days after spawning, OS: oak sawdust, OA: oak acorns, OLPR: olive pruning, VIP: vineyard pruning, Stage 1: mycelial run, stage 2: coat formation, stage 3: bump formation, stage 4: pigmentation, stage 5: coat hardening. Values are means ± SD. Different letters in the same column indicate a statistically significant difference according to Duncan's multiple range test at *P value <0*.*05*.

Depending on the shiitake strain under investigation, the duration of the spawn run phase on oak sawdust ranges from about 30 to 120 days after spawning [46]. Under the current experimental conditions, it ranged between 62.7 and 83.5 days after spawning for the strain 3782 cultivated on various substrates. Earlier, Özçelik and Pekşen [47] reported a different range (80–90 days) for shiitake cultivated on hazelnut husks.

Throughout the spawn run period, *L. edodes* degrades the lignocellulosic components of the substrate by secreting extracellular enzymes such as cellulase, hemicellulase, and ligninase [48]. Lignin, existing in the cell wall of the lignocellulosic material, protects cellulose and hemicellulose, making them resistant to enzymatic degradation [49]. Therefore, mushrooms degrade holocellulose (hemicellulose and cellulose) only after decomposing lignin [50,51]. The higher the proportion of lignin in the initial substrate mixture, the lower the substrate's bioavailability [19,52]. Further, shiitake mushrooms prefer a slightly acidic pH ranging from about 5.5 to 6.5 during spawn run and from about 4.5 to 5.5 during fruit formation [13,46]. Based on the previous statements, the current study showed that oak acorns, which had a similar pH, lower lignin content, and lower holocellulose (hemicellulose + cellulose) content compared to oak sawdust, caused the fastest mycelium development. Also, faster mycelia run and fruit formation obtained on OA-VIP:1-1 and OA-OLPR:1-1 compared to OA and OLPR can be attributed to the presence of OA in these substrates, especially since shiitake growth and fruiting were delayed by 20.8 and 12.1 days on OLPR and by 21.8 and 12.9 days on VIP compared to OA.

Further, earlier mycelia run obtained on OA-VIP:1-1 and OS-VIP:1-1 compared to VIP may be attributed to the lower holocellulose content and pH of the former substrates compared to the latter. On the contrary, Morales et al. [21] found earlier that vineyard pruning decreased the time needed for full mycelia colonization of *Ganoderma* spp. compared to oak sawdust and the mixture of oak sawdust and vineyard pruning in different ratios. Moreover, compared to the oak sawdust that is typically used for commercial production, an earlier harvest of 9.3 days from OA and 6.7 days from OA-VIP:1-1 offers considerable economic benefits by reducing the entire period of the production cycle of shiitake strain 3782.

On the other hand, the slowest mycelial development obtained on OLPR and OS-OLPR:1-1 could be explained by the high pH of both substrates (7.2 and 6.8, respectively). Although mycelia run was faster in OA-OLPR:1-1 and OS-OLPR:1-1 compared with OLPR, it was slower than that observed on oak sawdust. It can be consequently assumed that the high pH of OLPR negatively affected mycelia development in the substrate where it was present. On the contrary, Abou Fayssal et al. [19] found that the addition of low proportions of OLPR accelerated the mycelia run and shortened the production cycle of oyster mushrooms (*Pleurotus ostreatus*).

### 3.2 Shiitake production on tested substrates

Results in Table 3 showed that at the level of flushes 1 and 2, the average mushroom number was significantly reduced compared to control, except in OA (higher than OS) and in OA-VIP:1-1 (comparable to OS). Concerning mushroom weight recorded at flush 1, it showed a significant increase compared to control in all substrates, except in OA and OA-VIP:1-1, where it was comparable. At the level of flush 2, mushroom weight was comparable to control in all substrates, except VIP and OS-VIP:1-1, where it significantly increased. The biological yield recorded at flush 1 was comparable to control, except in VIP, OS-OLPR:1-1, and OS-VIP:1-1, where it showed a significant decrease (by 166.6, 74.4, and 54.6 g/kg compared to OS). On the other hand, the biological yield recorded at flush 2 was significantly lower than control in all substrates, except OA-VIP:1-1, where it was comparable (322.0 and 303.0 g/kg in OS and OA-VIP:1-1, respectively).

**Table 3:** Productive indicators at flush 1 (F1) and flush 2 (F2) recorded in tested substrates.

Substrates	MNF1	MNF2	MWF1 (g)	MWF2 (g)	BYF1 (g/kg)	BYF2 (g/kg)
**OS (control)**	29.5 ± 3.5 b	24.0 ± 2.8 b	8.8 ± 0.5c	11.6 ± 3.3bcd	275.0 ± 52.2 ab	322.0 ± 43.7a
**OA**	35.2 ± 3.1a	30.5 ± 1.5a	8.1 ± 1.5c	9.0 ± 2.6 d	282.8 ± 27.1 ab	269.2 ± 28.7 b
**OLPR**	16.7 ± 3.9cd	14.1 ± 2.7c	14.1 ± 0.5 b	12.3 ± 1.0BCE	320.3 ± 67.3a	168.0 ± 26.2 d
**VIP**	6.2 ± 0.8e	5.8 ± 2.1e	18.2 ± 2.0a	16.8 ± 3.5a	108.4 ± 3.1e	90.7 ± 2.3e
**OA-OLPR:1**–**1**	20.4 ± 4.7c	13.8 ± 2.3c	17.8 ± 2.6a	13.8 ± 1.9 b	319.4 ± 39.5a	212.9 ± 19.1c
**OA-VIP:1**–**1**	29.3 ± 3.4 b	22.8 ± 3.4 b	7.9 ± 0.9c	13.1 ± 0.5BCE	253.2 ± 37.6BCE	303.0 ± 39.5a
**OS-OLPR:1**–**1**	14.8 ± 3.2 d	10.8 ± 3.8cd	13.5 ± 1.0 b	10.5 ± 1.6cd	200.6 ± 22.4 d	122.6 ± 23.4e
**OS-VIP:1**–**1**	12.7 ± 3.2 d	9.5 ± 2.9 d	16.8 ± 2.5a	19.3 ± 2.7a	220.4 ± 19.6cd	195.4 ± 19.6cd
*P value*	0.000*	0.000*	0.000*	0.000*	0.000*	0.000*

OS: oak sawdust, OA: oak acorns, OLPR: olive pruning, VIP: vineyard pruning, MN: mushroom number, MW: mushroom weight, BY: biological yield. Values are means ± SD. Different letters in the same column indicate a statistically significant difference according to Duncan's multiple range test at *P value <0*.*05*.

Furthermore, comparing both flushes, OA gave a significantly higher number of mushrooms compared to VIP, while the latter caused a significantly higher mushroom weight. Mixing OA and VIP improved the biological yields at flush 1 and 2 compared to VIP alone (by 144.6 and 212.3 g/kg, respectively) and the biological yield at flush 2 compared to OA alone (by 33.8 g/kg). On the other hand, mixing OA with OLPR improved the biological yield at the second flush compared to OLPR alone, but it significantly decreased it compared to OA alone. Mixing OS with OLPR or with VIP resulted in significantly lower biological yields at both flushes compared to OS, OLPR, and VIP alone. Also, in terms of biological yields, the substrate OA-OLPR: 1-1 was superior to OS-OLPR:1-1 at both flushes, while the substrate OA-VIP:1-1 exceeded OS-VIP:1-1 only at flush 2. This superiority could be attributed to the improvement in mushroom number because of the use of OA and the higher mushroom weight because of the use of VIP and OLPR. Mushroom weight ranged between 7.9 and 19.3 g on various substrates. Ranges of this indicator reported in earlier studies were higher: 14.9–33.5 g by Baktemur et al. [15], 12.9–19.4 g by Atila [53], 19.0–70 g by Martínez-Guerrero et al. [54], and 18.3–36.6 g by Annepu et al. [1].

**Fig. 3:**
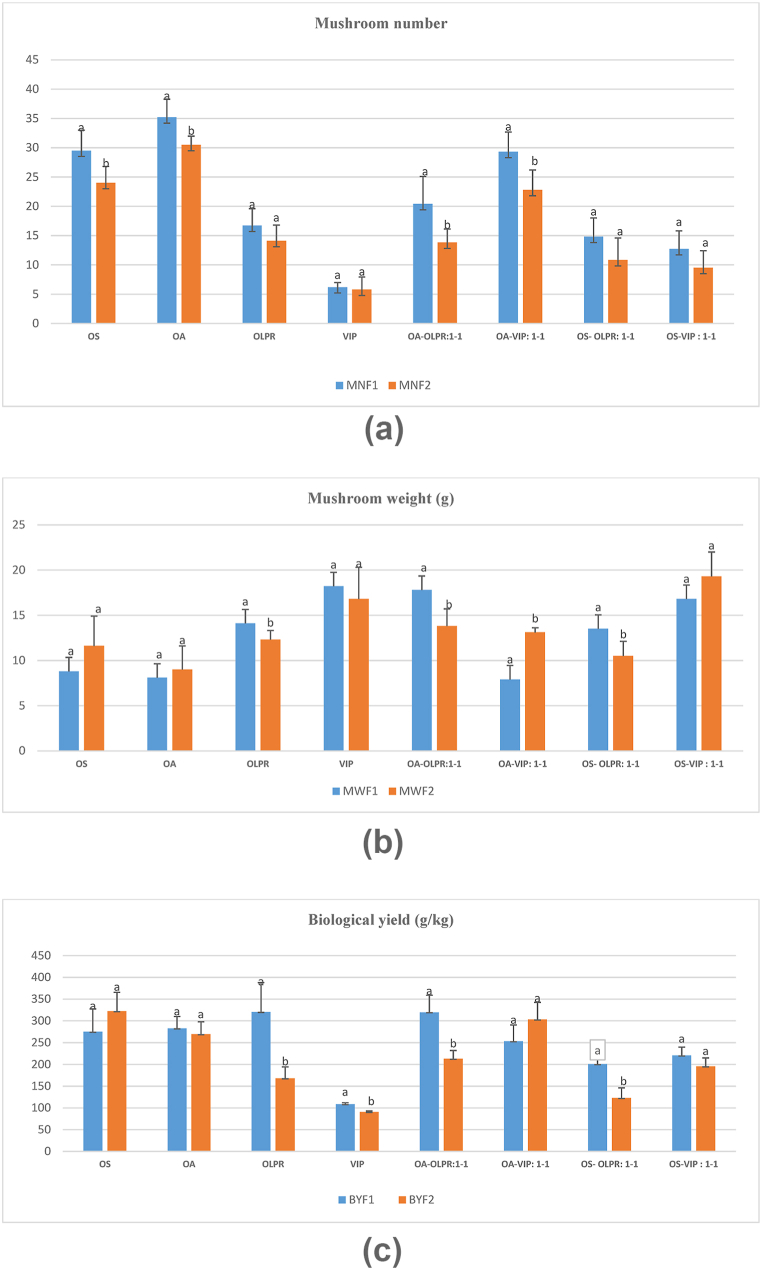
a Comparison of mushroom number obtained at flush 1 and flush 2 in each tested substrate. (OS: oak sawdust, OA: oak acorns, OLPR: olive pruning, VIP: vineyard pruning. Values are means ± SD. Different letters indicate a statistically significant difference at *P value* < 0.05 according to the independent sample *t*-test. b Comparison of mushroom weight (g) obtained at flush 1 and flush 2 in each tested substrate. c Comparison of biological yield (g/kg) obtained at flush 1 and flush 2 in each tested substrate.

The comparison of productive indicators between flush 1 and 2 showed that the mushroom number (Fig. 3a) significantly decreased in flush 2 in OS, OA, OA-OLPR:1-1, and OA-VIP:1-1 by 5.5, 4.7, 6.6, and 6.5 on average, respectively. Also, mushroom weight (Fig. 3b) significantly decreased in flush 2 in OLPR, OA-OLPR:1-1, and OS-OLPR:1-1 by 1.8, 4.0, and 3.0 g, respectively, while it increased in OA-VIP:1-1 by 5.2 g on average. Further, the biological yield (Fig. 3c) decreased significantly in flush 2 in OLPR, VIP, OA-OLPR:1-1, and OS-OLPR:1-1 by 152.3, 17.7, 106.5, and 78 g/kg, respectively. On the contrary, production was consistent (no significant difference) in OS, OA, OA-VIP:1-1, and OS-VIP:1-1 among consecutive flushes.

Moreover, the total biological yield (Table 4) was significantly reduced in OLPR, OS-OLPR:1-1, VIP, and OS-VIP:1-1 compared to OS by 18.0, 45.8, 66.6, and 30.3 %, respectively. On the other hand, the substrates OA, OA-OLPR:1-1, and OA-VIP:1-1 recorded comparable total biological yields compared to OS at 552.0, 532.2, and 556.2 g/kg, respectively, compared to 597.0 g/kg in OS.

**Table 4:** Total production obtained from tested substrates.

Substrates	Total biological yield (g/kg)	Biological efficiency (%)
**OS (control)**	597.0 ± 93.2a	59.7 ± 9.3a
**OA**	552.0 ± 53.1 ab	55.2 ± 5.3 ab
**OLPR**	488.3 ± 83.2 b	48.8 ± 8.3 b
**VIP**	199.1 ± 5.3e	19.9 ± 0.5e
**OA-OLPR:1**–**1**	532.3 ± 47.3 ab	53.2 ± 4.7 ab
**OA-VIP:1**–**1**	556.2 ± 76.9 ab	55.6 ± 7.7 ab
**OS-OLPR:1**–**1**	323.2 ± 45.5 d	32.3 ± 4.6 d
**OS-VIP:1**–**1**	415.8 ± 38.7c	41.6 ± 3.9c
*P value*	0.000*	0.000*


OS: oak sawdust, OA: oak acorns, OLPR: olive pruning, VIP: vineyard pruning, MN: mushroom number, MW: mushroom weight. Values are means ± SD. Different letters in the same column indicate a statistically significant difference according to Duncan's multiple range test at *P value <0*.*05*.

According to Chen [46], farmers can harvest 0.3–0.5 kg of fresh shiitake mushrooms from 1 kg of dried substrate (30–50 % BE). Biological yields obtained from the various tested substrates were comparable, except for VIP, and ranged between 0.42 on OS-VIP:1-1 and 0.59 kg on oak sawdust. On an experimental scale, Yu et al. [55] reported a higher biological efficiency on oak sawdust (80.8 %), and Leifa et al. [56] reported a different range of 78.4–85.8 % on various lignocellulosic substrates. Baktemur et al. [15] reported biological efficiency values of 45.0, 65.7, and 21.3 % on substrates composed of oak sawdust, VIP, and VIP-OS, respectively.

One crucial factor affecting the growth and fruiting of mushrooms is the C/N ratio of the growing substrate [18,57,58]. It was noted that a minimum of 25:1 and a maximum of 55:1 C/N ratios are required for shiitake growth [59]. Desisa et al. [12] found higher total yields of shiitake on substrates with a low C/N (20.96). However, in the current experiment, higher total yields were obtained on substrates with higher C/N ratios.

### 3.3 Physical characteristics of shiitake mushrooms

Evaluation of mushrooms’ physical characteristics (Table 5) showed that in comparison to control, there was a superiority in terms of mushroom firmness in OA and OS-OLPR:1-1 (increase by 1.4 and 2.0 mm, respectively), pileus diameter in OS-OLPR:1-1 (increase by 1.6 cm), pileus thickness in OA-VIP:1-1 (increase by 0.5 cm), stipe diameter in OS-VIP:1-1 (increase by 0.2 cm), and the ratio PD/SL in OS-VIP:1-1 and OS-OLPR:1-1. Earlier, Moonmoon et al. [60] reported the following characteristics for shiitake mushrooms cultivated on oak sawdust: pileus diameter of 6.0 cm, pileus thickness of 1.0 cm, stipe length of 4.5 cm, and stipe diameter of 1.0 cm. Also, Baktemur et al. [15] obtained the following ranges: 4.5–6.1 cm, 0.8–2.7 cm, and 2.1–4.9 cm, for the pileus diameter, stipe diameter, and stipe length, respectively.

**Table 5:** Mushrooms’ physical characteristics recorded at tested substrates.

Substrates	Firmness (mm)	PD (cm)	PT (cm)	SD (cm)	SL (cm)	PD/SL
**OS (control)**	5.2 ± 0.8BCE	5.3 ± 1.3 b	1.2 ± 0.01c	0.9 ± 0.2BCE	3.2 ± 0.8a	1.9 ± 0.4 b
**OA**	6.6 ± 0.8a	4.4 ± 0.5 b	1.1 ± 0.1c	0.7 ± 0.1 d	2.3 ± 0.3cd	2.1 ± 0.2 ab
**OLPR**	4.4 ± 0.6c	5.0 ± 0.2 b	1.3 ± 0.1BCE	1.0 ± 0.1 ab	2.5 ± 0.3BCE	2.1 ± 0.4 ab
**VIP**	5.1 ± 1.3BCE	5.4 ± 0.2 b	1.5 ± 0.1 b	0.8 ± 0.1cd	2.6 ± 0.5BCE	2.1 ± 0.3 ab
**OA-OLPR:1**–**1**	5.5 ± 0.6 b	5.1 ± 0.5 b	1.3 ± 0.1BCE	0.9 ± 0.1 ab	2.6 ± 0.2BCE	2.3 ± 0.5 ab
**OA-VIP:1**–**1**	5.5 ± 0.1 b	4.9 ± 0.4 b	1.7 ± 0.1a	0.7 ± 0.1 d	2.5 ± 0.3BCE	2.03 ± 0.2 b
**OS-OLPR:1**–**1**	7.2 ± 0.4a	6.9 ± 2.6a	1.3 ± 0.0BCE	0.9 ± 0.2BCE	2.8 ± 0.04 ab	2.5 ± 0.4a
**OS-VIP:1**–**1**	5.5 ± 0.9 b	4.6 ± 0.8 b	1.2 ± 0.5BCE	1.1 ± 0.2a	1.9 ± 0.5 d	2.5 ± 0.3a
*P value*	0.000*	0.015	0.000*	0.000*	0.000*	0.028

OS: oak sawdust, OA: oak acorns, OLPR: olive pruning, VIP: vineyard pruning, PD: pileus diameter, PT: pileus thickness, SD: stipe diameter, SL: stipe length. Values are means ± SD. Different letters in the same column indicate a statistically significant difference according to Duncan's multiple range test at *P value <0*.*05*.

Firmness is an important quality parameter in overall product acceptance for fresh shiitake mushrooms [61]. More firm mushrooms are preferable at the market and have a longer shelf life. Furthermore, mushrooms with a greater PD/SL ratio have higher marketability. Larger mushrooms are more expensive since they have thicker and bigger pilei with shorter stipes. While the stipe is too difficult to consume and is typically eliminated, the pileus provides the mushroom with a more substantial bite, especially if it is intended to be eaten whole [62].

### 3.4 Correlation between productive indicators

Results of stepwise regression (Table 6) showed that variations in the total biological yield (TBY) in OS, OLPR, VIP, and OS-VIP:1-1 were the most strongly influenced by the average biological yield obtained at the first flush (BYF1) (R^2^ = 0.94, 0.90, 0.95, and 0.97, respectively). Such correlations were positive for the first three substrates and negative for the fourth one. In OA-OLPR:1-1, TBY was the most positively affected by both BYF1 and PD (pileus diameter recorded at the first flush) (R^2^ = 0.98). In OS-OLPR:1-1, a higher mushroom weight recorded in the first flush was negatively correlated with TBY (R^2^ = 0.98) obtained in this substrate. In OA-VIP:1-1 a higher biological yield recorded at the second flush was negatively correlated with TBY (R^2^ = 0.995).

**Table 6:** Significant correlations between the total biological yield (dependent variable) and productive indicators (independent variables) in the tested treatments (n = _6)_.

Substrates	dependent	independent	equation	Adj R^2^
**OS (control)**	TBY	BYF1	117.349 + (0.977 x BYF1)	0.94
**OA**	TBY	BYF2	76.97+ (0.954 x BYF2)	0.88
**OLPR**	TBY	BYF1	107.45 + (0.961x BYF1)	0.90
**VIP**	TBY	BYF1	17.732 + (0.981 x BYF1)	0.95
**OA-OLPR:1**–**1**	TBY	BYF1	156.888 + (0.981 x BYF1)	0.95
		BYF1, PD	90.490+ (0.88 x BYF1) + (0.201 x PD)	0.98
**OA-VIP:1**–**1**	TBY	BYF2	−32.252 +(0.997 x BYF2)	0.99
**OS-OLPR:1**–**1**	TBY	MWF1	−320.043 + (0.995 x FWF1)	0.98
**OS-VIP:1**–**1**	TBY	BYF1	−13.648 + (0.988 x BYF1)	0.97

OS: oak sawdust, OA: oak acorns, OLPR: olive pruning, VIP: vineyard pruning, TBY: total biological yield, BYF1: biological yield at flush 1, BYF2: biological yield at flush 2, PD: pileus diameter, MWF1: mushroom weight at flush 1.

### 3.5 Nutritional composition of shiitake mushrooms

The nutritional composition of mushrooms is highly affected by the composition of substrates used to grow them [[63], [64], [65]] and the strain under cultivation [66]. The analysis of mushroom composition (Table 7) showed that the mushrooms’ protein content was significantly higher on all tested substrates compared to OS. Also, it was significantly improved in substrates containing OLPR (OLPR, OA-OLPR:1-1, and OS-OLPR:1-1) compared to the other substrates, with the highest value recorded in OA-OLPR:1-1 (8.7 %). Protein content was significantly higher in VIP compared with OS, OA, OA-VIP:1-1, and OS-VIP:1-1 (5.8, 2.7, 4.8, 5.3, and 3.2 %, respectively), OA compared to OS (4.8 and 2.7 %, respectively), OA-VIP:1-1 compared to OS-VIP:1-1 (5.3 and 3.2 %, respectively), and OA-OLPR:1-1 compared to OS-OLPR:1-1 (8.7 and 8.0 %, respectively). The protein content of shiitake 3782 ranged between 2.7 % and 8.7 %. This range was lower than those mentioned by Desisa et al. [12]: 13.7–19.6 %, Gaitán-Hernández et al. [20]: 12.4–17.2 %, and Rahman and Choudhury [67]: 20–23 % for different shiitake strains cultivated on various agricultural wastes. Mushrooms are regarded as a valuable source of protein from a nutritional point of view. This is in line with the assumption that mushrooms may effectively replace meat, and their nutritional value is comparable to that of many plant species [68]. Efforts are being made nowadays to search for protein sources to fill the nutritional needs of a growing world population [69]. The use of all tested substrates provides a suitable method for the valorization of agro-forest wastes through the production of protein-rich shiitake mushrooms.

**Table 7:** Composition of Shiitake mushrooms cultivated on tested substrates.

Substrates	Proteins (%)	Fibers (%)	Fat (%)	Ash (%)	Carbs	Vit C (mg/100 g)	Vit D (μg/L)
**OS (control)**	2.7 ± 0.2g	1.5 ± 0.1f	0.28 ± 0.02a	0.6 ± 0.06 d	7.3 ± 0.1c	4.5 ± 0.1de	<3
**OA**	4.8 ± 0.1e	1.8 ± 0.1e	0.31 ± 0.02a	1.0 ± 0.10BCE	6.4 ± 0.2 d	3.9 ± 0.1f	<3
**OLPR**	8.5 ± 0.3a	2.8 ± 0.1a	0.31 ± 0.08a	0.9 ± 0.05BCE	3.0 ± 0.3 g	7.0 ± 0.2a	<3
**VIP**	5.8 ± 0.3c	1.8 ± 0.2e	0.30 ± 0.01a	1.6 ± 0.10a	19.2 ± 0.2a	4.0 ± 0.6ef	<3
**OA-OLPR:1**–**1**	8.7 ± 0.1a	2.1 ± 0.1BCE	0.32 ± 0.02a	1.0 ± 0.08BCE	3.7 ± 0.4f	6.0 ± 0.4 b	<3
**OA-VIP:1**–**1**	5.3 ± 0.2 d	2.3 ± 0.2 b	0.29 ± 0.04a	1.1 ± 0.20 b	6.6 ± 0.6 d	4.1 ± 0.2ef	<3
**OS-OLPR:1**–**1**	8.0 ± 0.1 b	1.9 ± 0.05de	0.28 ± 0.06a	1.0 ± 0.09BCE	4.9 ± 0.5e	5.0 ± 0.5c	<3
**OS-VIP:1**–**1**	3.2 ± 0.1f	2.1 ± 0.1cd	0.30 ± 0.05a	0.8 ± 0.10cd	8.2 ± 0.1 b	4.8 ± 0.1cd	<3
*P value*	0.000*	0.000*	0.000*	0.000*	0.000*	0.000*	–

OS: oak sawdust, OA: oak acorns, OLPR: olive pruning, VIP: vineyard pruning, carbs: carbohydrates. Values are means ± SD. Different letters in the same column indicate a statistically significant difference according to Duncan's multiple range test at *P value <0*.*05*.

Mushrooms’ fiber content was ameliorated in all tested substrates compared to OS and was the highest in OLPR (2.8 %). This component was also significantly higher in OA-VIP:1-1 compared to OA, VIP, and OS-VIP:1-1 (2.3, 1.8, 1.8, and 2.1 %, respectively). Twenty-five percent of the daily required intake of dietary fibre can be obtained from eating edible mushrooms [70]. Mushroom fibers, such as chitin and β-glucans, combat obesity, diabetes, and hypertension and have many applications in biomedicine because of their anti-inflammatory, anti-allergic, anticancer, and immunomodulatory effects [71,72]. Ash content was higher in mushrooms of all substrates, except OS-VIP: 1-1, compared to OS, with the highest value in VIP (1.6 %). Carbohydrate content was significantly lower in most substrates compared to OS, except in VIP and OS-VIP:1-1, where it was significantly higher. Vitamin C content was ameliorated in mushrooms of OLPR, OA-OLPR:1-1, and OS-OLPR compared to OS and OA (7.0, 6.0, 5.0, 4.5, and 3.9 mg/100g, respectively). Finally, vitamin D content of mushrooms was not affected by the substrate composition (<3 μg/L on all substrates).

## 4 Conclusion

- “Conclusively, the substrates OA, OA-VIP:1-1, and OA-OLPR:1-1 are recommended as alternatives to the standard oak sawdust substrate. The use of these substrates at an experimental level showed promising results that might be applied at an industrial scale for producing shiitake.
- OA, OA-VIP:1-1, and OA-OLPR:1-1 had greater protein and fiber content, offered similar yields and improved the nutritional value of the produced mushrooms in comparison to the standard substrate.
- OA, and OA-VIP:1-1 allowed a high and continuous production over two consecutive flushes and shortened the production cycle in comparison to the oak sawdust. Thus, they might be more favorable than OA-OLPR:1-1.
- The main limitation of using OA, VIP and OLP in shiitake cultivation may be their limited availability in some countries where oak, olive, and vines are not present.
- Future studies in this field may assess the effect of vineyard and olive pruning on the flavor of the shiitake mushrooms. They may also investigate supplementing the substrate OA-OLPR: 1-1 using nitrogenous additive as a method to overcome the delay in harvest and the decline in production at the second flush in such substrate.

## Funding

This work was supported by the BULGARIAN DEVELOPMENT AID, Grant no. 1.

## Data availability statement

The data associated with our study wasn't deposited into a publicly available repository but it is uploaded as a supplementary material.

## CRediT authorship contribution statement

**Youssef Najib Sassine:** Validation, Supervision, Resources, Project administration, Methodology, Funding acquisition. **Stephanie Nabhan:** Writing – original draft, Software, Investigation, Formal analysis, Data curation. **Elina Rachkidy:** Writing – original draft, Investigation, Data curation. **Zeina El Sebaaly:** Writing – review & editing, Visualization, Validation, Software, Project administration, Methodology, Formal analysis, Conceptualization.

## Declaration of competing interest

All other authors declare that they have no known competing interests that could have appeared to influence the work reported in this paper.
