# Supplementary material for: Valorization of agro-forest wastes (oak acorns, vineyard pruning, and olive pruning) through the cultivation of shiitake (Lentinula edodes) mushrooms
Source: Heliyon. 2024 Jun 15;10(12):e32562. doi: 10.1016/j.heliyon.2024.e32562 (PMC11237938; doi:10.1016/j.heliyon.2024.e32562)
Supplement: Multimedia component 1 [file mmc1.docx]

Date used in the Table 2

| Treatment | stage 1 | stage2 | stage3 | stage4 | stage5 | fruiting | Harvest |
| --- | --- | --- | --- | --- | --- | --- | --- |
| OS-WB: 800-200 | 16.00 | 21.00 | 35.00 | 58.00 | 70.00 | 75.00 | 79.00 |
| OS-WB: 800-200 | 15.00 | 25.00 | 41.00 | 64.00 | 73.00 | 76.00 | 80.00 |
| OS-WB: 800-200 | 16.00 | 24.00 | 35.00 | 60.00 | 74.00 | 77.00 | 82.00 |
| OS-WB: 800-200 | 17.00 | 20.00 | 40.00 | 63.00 | 71.00 | 74.00 | 78.00 |
| OS-WB: 800-200 | 14.00 | 24.00 | 38.00 | 65.00 | 70.00 | 74.00 | 78.00 |
| OS-WB: 800-200 | 16.00 | 23.00 | 35.00 | 58.00 | 75.00 | 77.00 | 80.00 |
| OA-WB: 800-200 | 13.00 | 21.00 | 33.00 | 48.00 | 59.00 | 62.00 | 66.00 |
| OA-WB: 800-200 | 13.00 | 20.00 | 33.00 | 50.00 | 57.00 | 61.00 | 66.00 |
| OA-WB: 800-200 | 14.00 | 23.00 | 36.00 | 59.00 | 68.00 | 71.00 | 75.00 |
| OA-WB: 800-200 | 16.00 | 24.00 | 37.00 | 59.00 | 69.00 | 71.00 | 76.00 |
| OA-WB: 800-200 | 10.00 | 19.00 | 32.00 | 53.00 | 61.00 | 65.00 | 69.00 |
| OA-WB: 800-200 | 10.00 | 17.00 | 31.00 | 54.00 | 62.00 | 65.00 | 69.00 |
| OLP-WB: 800-200 | 11.20 | 23.00 | 40.00 | 66.00 | 80.10 | 84.50 | 89.00 |
| OLP-WB: 800-200 | 21.80 | 33.40 | 49.00 | 76.00 | 86.60 | 90.10 | 94.00 |
| OLP-WB: 800-200 | 20.20 | 30.00 | 50.00 | 76.00 | 85.80 | 89.80 | 94.00 |
| OLP-WB: 800-200 | 15.00 | 22.50 | 37.20 | 68.20 | 82.00 | 85.90 | 91.00 |
| OLP-WB: 800-200 | 12.60 | 23.20 | 39.60 | 67.60 | 78.80 | 82.90 | 87.00 |
| OLP-WB: 800-200 | 23.90 | 32.50 | 47.00 | 77.00 | 87.60 | 91.40 | 96.00 |
| OA-OLPR-WB: 400-400-200 | 11.00 | 21.00 | 39.00 | 62.00 | 76.00 | 79.00 | 83.00 |
| OA-OLPR-WB: 400-400-200 | 17.00 | 28.00 | 38.00 | 62.00 | 73.00 | 78.00 | 82.00 |
| OA-OLPR-WB: 400-400-200 | 16.00 | 28.00 | 41.00 | 68.00 | 76.00 | 79.00 | 83.00 |
| OA-OLPR-WB: 400-400-200 | 11.00 | 20.00 | 35.00 | 62.00 | 71.00 | 75.00 | 80.00 |
| OA-OLPR-WB: 400-400-200 | 12.00 | 22.00 | 38.00 | 63.00 | 74.00 | 77.00 | 81.00 |
| OA-OLPR-WB: 400-400-200 | 14.00 | 23.00 | 38.00 | 62.00 | 74.00 | 77.00 | 82.00 |
| OS-OA-OLPR-WB: 267-267-267-200 | 10.00 | 20.00 | 35.00 | 65.00 | 78.00 | 82.00 | 88.00 |
| OS-OA-OLPR-WB: 267-267-267-200 | 17.00 | 26.00 | 40.00 | 68.00 | 79.00 | 82.00 | 87.00 |
| OS-OA-OLPR-WB: 267-267-267-200 | 14.00 | 23.00 | 38.00 | 66.00 | 80.00 | 84.00 | 89.00 |
| OS-OA-OLPR-WB: 267-267-267-200 | 17.00 | 28.00 | 40.00 | 71.00 | 81.00 | 84.00 | 88.00 |
| OS-OA-OLPR-WB: 267-267-267-200 | 13.00 | 21.00 | 36.00 | 65.00 | 78.00 | 81.00 | 86.00 |
| OS-OA-OLPR-WB: 267-267-267-200 | 16.00 | 26.00 | 40.00 | 68.00 | 79.00 | 83.00 | 87.00 |
| VIP-WB: 800-200 | 13.00 | 23.00 | 40.00 | 66.00 | 76.00 | 79.00 | 83.00 |
| VIP-WB: 800-200 | 15.00 | 21.00 | 36.00 | 62.00 | 74.00 | 77.00 | 81.00 |
| VIP-WB: 800-200 | 16.00 | 23.00 | 37.00 | 64.00 | 74.00 | 78.00 | 82.00 |
| VIP-WB: 800-200 | 14.00 | 21.00 | 36.00 | 64.00 | 75.00 | 80.00 | 84.00 |
| VIP-WB: 800-200 | 13.00 | 23.00 | 39.00 | 65.00 | 75.00 | 79.00 | 84.00 |
| VIP-WB: 800-200 | 15.00 | 22.00 | 38.00 | 65.00 | 75.00 | 79.00 | 85.00 |
| OA-VIP-WB: 400-400-200 | 14.00 | 21.00 | 33.00 | 55.00 | 65.00 | 68.00 | 72.00 |
| OA-VIP-WB: 400-400-200 | 10.00 | 18.00 | 32.00 | 52.00 | 61.00 | 66.00 | 72.00 |
| OA-VIP-WB: 400-400-200 | 14.00 | 22.00 | 34.00 | 56.00 | 66.00 | 70.00 | 74.00 |
| OA-VIP-WB: 400-400-200 | 15.00 | 22.00 | 36.00 | 56.00 | 65.00 | 68.00 | 73.00 |
| OA-VIP-WB: 400-400-200 | 15.00 | 23.00 | 35.00 | 57.00 | 65.00 | 68.00 | 74.00 |
| OA-VIP-WB: 400-400-200 | 12.00 | 19.00 | 32.00 | 54.00 | 64.00 | 68.00 | 72.00 |
| OS-OA-VIP-WB: 267-267-267-200) | 11.00 | 18.00 | 33.00 | 56.00 | 66.00 | 69.00 | 75.00 |
| OS-OA-VIP-WB: 267-267-267-200) | 13.00 | 21.00 | 35.00 | 58.00 | 68.00 | 71.00 | 76.00 |
| OS-OA-VIP-WB: 267-267-267-200) | 12.00 | 19.00 | 33.00 | 55.00 | 66.00 | 70.00 | 75.00 |
| OS-OA-VIP-WB: 267-267-267-200) | 12.00 | 19.00 | 35.00 | 59.00 | 69.00 | 72.00 | 76.00 |
| OS-OA-VIP-WB: 267-267-267-200) | 12.00 | 20.00 | 36.00 | 58.00 | 68.00 | 72.00 | 77.00 |
| OS-OA-VIP-WB: 267-267-267-200) | 16.00 | 23.00 | 37.00 | 60.00 | 71.00 | 74.00 | 78.00 |

Data used in the Table 3 and Table 6

| Treatment | MN F1 | MW F1 | BY F1 | MN F2 | MW F2 | BYF2 |
| --- | --- | --- | --- | --- | --- | --- |
| OS-WB: 800-200 | 36.00 | 8.20 | 202.90 | 29.00 | 8.09 | 290.30 |
| OS-WB: 800-200 | 31.00 | 8.40 | 345.20 | 21.00 | 16.70 | 390.70 |
| OS-WB: 800-200 | 28.00 | 8.80 | 320.50 | 22.00 | 14.50 | 343.50 |
| OS-WB: 800-200 | 27.00 | 9.10 | 240.50 | 25.00 | 9.30 | 280.50 |
| OS-WB: 800-200 | 27.00 | 9.70 | 280.60 | 23.00 | 10.80 | 340.60 |
| OS-WB: 800-200 | 28.00 | 8.60 | 260.50 | 24.00 | 10.31 | 286.30 |
| OA-WB: 800-200 | 38.00 | 6.80 | 290.50 | 28.50 | 12.30 | 285.50 |
| OA-WB: 800-200 | 36.00 | 9.00 | 256.20 | 29.20 | 6.30 | 250.00 |
| OA-WB: 800-200 | 37.00 | 6.83 | 248.60 | 31.20 | 7.50 | 246.20 |
| OA-WB: 800-200 | 30.00 | 10.20 | 320.50 | 32.30 | 9.80 | 298.30 |
| OA-WB: 800-200 | 33.00 | 9.00 | 300.60 | 31.50 | 6.50 | 300.00 |
| OA-WB: 800-200 | 37.00 | 6.83 | 280.50 | 30.10 | 11.50 | 235.20 |
| OLP-WB: 800-200 | 23.40 | 13.90 | 370.00 | 13.50 | 13.50 | 174.82 |
| OLP-WB: 800-200 | 13.00 | 14.50 | 219.80 | 15.70 | 10.80 | 150.60 |
| OLP-WB: 800-200 | 18.88 | 13.80 | 260.60 | 13.87 | 12.60 | 174.70 |
| OLP-WB: 800-200 | 16.60 | 14.90 | 383.60 | 18.20 | 12.30 | 210.40 |
| OLP-WB: 800-200 | 13.90 | 13.50 | 370.00 | 13.20 | 11.30 | 164.28 |
| OLP-WB: 800-200 | 14.50 | 14.10 | 317.80 | 10.16 | 13.10 | 133.06 |
| OA-OLPR-WB: 400-400-200 | 19.00 | 20.60 | 334.60 | 15.00 | 12.80 | 232.60 |
| OA-OLPR-WB: 400-400-200 | 21.00 | 16.20 | 314.90 | 13.00 | 13.30 | 217.30 |
| OA-OLPR-WB: 400-400-200 | 20.20 | 17.30 | 320.20 | 12.00 | 14.60 | 203.60 |
| OA-OLPR-WB: 400-400-200 | 13.20 | 21.50 | 261.00 | 13.00 | 14.40 | 180.50 |
| OA-OLPR-WB: 400-400-200 | 21.07 | 15.90 | 303.90 | 18.00 | 10.90 | 213.90 |
| OA-OLPR-WB: 400-400-200 | 28.00 | 15.50 | 381.80 | 12.00 | 16.60 | 229.40 |
| OS-OA-OLPR-WB: 267-267-267-200 | 19.00 | 12.65 | 175.50 | 10.00 | 10.50 | 101.21 |
| OS-OA-OLPR-WB: 267-267-267-200 | 12.00 | 14.39 | 225.90 | 8.00 | 11.60 | 143.89 |
| OS-OA-OLPR-WB: 267-267-267-200 | 15.00 | 12.65 | 180.40 | 15.00 | 8.90 | 101.21 |
| OS-OA-OLPR-WB: 267-267-267-200 | 14.00 | 14.39 | 217.60 | 9.00 | 11.10 | 143.89 |
| OS-OA-OLPR-WB: 267-267-267-200 | 18.00 | 12.65 | 185.80 | 7.00 | 12.30 | 101.21 |
| OS-OA-OLPR-WB: 267-267-267-200 | 11.00 | 14.39 | 218.50 | 16.00 | 8.30 | 143.89 |
| VIP-WB: 800-200 | 6.00 | 19.75 | 108.67 | 6.00 | 16.50 | 91.54 |
| VIP-WB: 800-200 | 7.00 | 14.77 | 103.30 | 3.00 | 22.30 | 87.90 |
| VIP-WB: 800-200 | 6.00 | 18.22 | 109.32 | 6.00 | 15.20 | 89.59 |
| VIP-WB: 800-200 | 7.00 | 17.73 | 106.38 | 4.00 | 19.16 | 88.67 |
| VIP-WB: 800-200 | 5.00 | 20.40 | 111.56 | 9.00 | 12.60 | 93.67 |
| VIP-WB: 800-200 | 6.00 | 18.50 | 110.98 | 7.00 | 14.80 | 92.82 |
| OA-VIP-WB: 400-400-200 | 36.00 | 6.51 | 225.32 | 24.00 | 12.92 | 278.32 |
| OA-VIP-WB: 400-400-200 | 29.00 | 7.44 | 220.84 | 28.00 | 12.43 | 274.98 |
| OA-VIP-WB: 400-400-200 | 27.00 | 9.02 | 315.45 | 19.00 | 13.71 | 368.44 |
| OA-VIP-WB: 400-400-200 | 29.00 | 7.95 | 247.93 | 20.00 | 13.43 | 287.86 |
| OA-VIP-WB: 400-400-200 | 27.00 | 8.46 | 280.85 | 21.00 | 13.25 | 334.69 |
| OA-VIP-WB: 400-400-200 | 28.00 | 8.31 | 229.08 | 25.00 | 12.77 | 273.38 |
| OS-OA-VIP-WB: 267-267-267-200) | 9.00 | 20.12 | 198.84 | 11.00 | 16.50 | 170.50 |
| OS-OA-VIP-WB: 267-267-267-200) | 17.00 | 13.30 | 202.60 | 6.00 | 22.40 | 183.10 |
| OS-OA-VIP-WB: 267-267-267-200) | 14.00 | 16.40 | 225.20 | 9.00 | 20.90 | 197.20 |
| OS-OA-VIP-WB: 267-267-267-200) | 15.00 | 14.70 | 211.70 | 7.00 | 21.30 | 192.60 |
| OS-OA-VIP-WB: 267-267-267-200) | 11.00 | 17.80 | 249.50 | 10.00 | 18.80 | 228.70 |
| OS-OA-VIP-WB: 267-267-267-200) | 10.00 | 18.50 | 234.50 | 14.00 | 15.90 | 200.30 |

Data used in the Table 4 and Table 6

| Treatment | TBY | BE |
| --- | --- | --- |
| OS-WB: 800-200 | 493.20 | 49.32 |
| OS-WB: 800-200 | 735.90 | 73.59 |
| OS-WB: 800-200 | 664.00 | 66.40 |
| OS-WB: 800-200 | 521.00 | 52.10 |
| OS-WB: 800-200 | 621.20 | 62.12 |
| OS-WB: 800-200 | 546.80 | 54.68 |
| OA-WB: 800-200 | 576.00 | 57.60 |
| OA-WB: 800-200 | 506.20 | 50.62 |
| OA-WB: 800-200 | 494.80 | 49.48 |
| OA-WB: 800-200 | 618.80 | 61.88 |
| OA-WB: 800-200 | 600.60 | 60.06 |
| OA-WB: 800-200 | 515.70 | 51.57 |
| OLP-WB: 800-200 | 544.82 | 54.48 |
| OLP-WB: 800-200 | 370.40 | 37.04 |
| OLP-WB: 800-200 | 435.30 | 43.53 |
| OLP-WB: 800-200 | 594.00 | 59.40 |
| OLP-WB: 800-200 | 534.28 | 53.43 |
| OLP-WB: 800-200 | 450.86 | 45.09 |
| OA-OLPR-WB: 400-400-200 | 566.20 | 56.62 |
| OA-OLPR-WB: 400-400-200 | 532.20 | 53.22 |
| OA-OLPR-WB: 400-400-200 | 525.00 | 52.50 |
| OA-OLPR-WB: 400-400-200 | 459.50 | 45.95 |
| OA-OLPR-WB: 400-400-200 | 512.80 | 51.28 |
| OA-OLPR-WB: 400-400-200 | 598.00 | 59.80 |
| OS-OA-OLPR-WB: 267-267-267-200 | 276.71 | 27.67 |
| OS-OA-OLPR-WB: 267-267-267-200 | 369.79 | 36.98 |
| OS-OA-OLPR-WB: 267-267-267-200 | 281.61 | 28.16 |
| OS-OA-OLPR-WB: 267-267-267-200 | 361.49 | 36.15 |
| OS-OA-OLPR-WB: 267-267-267-200 | 287.01 | 28.70 |
| OS-OA-OLPR-WB: 267-267-267-200 | 362.39 | 36.24 |
| VIP-WB: 800-200 | 200.21 | 20.02 |
| VIP-WB: 800-200 | 191.27 | 19.13 |
| VIP-WB: 800-200 | 198.91 | 19.89 |
| VIP-WB: 800-200 | 195.05 | 19.51 |
| VIP-WB: 800-200 | 205.23 | 20.52 |
| VIP-WB: 800-200 | 203.80 | 20.38 |
| OA-VIP-WB: 400-400-200 | 503.64 | 50.36 |
| OA-VIP-WB: 400-400-200 | 495.82 | 49.58 |
| OA-VIP-WB: 400-400-200 | 683.89 | 68.39 |
| OA-VIP-WB: 400-400-200 | 535.79 | 53.58 |
| OA-VIP-WB: 400-400-200 | 615.54 | 61.55 |
| OA-VIP-WB: 400-400-200 | 502.46 | 50.25 |
| OS-OA-VIP-WB: 267-267-267-200) | 369.34 | 36.93 |
| OS-OA-VIP-WB: 267-267-267-200) | 385.70 | 38.57 |
| OS-OA-VIP-WB: 267-267-267-200) | 422.40 | 42.24 |
| OS-OA-VIP-WB: 267-267-267-200) | 404.30 | 40.43 |
| OS-OA-VIP-WB: 267-267-267-200) | 478.20 | 47.82 |
| OS-OA-VIP-WB: 267-267-267-200) | 434.80 | 43.48 |

Data used in the Table 5 and Table 6

| Treatment | PD F1 | PT F1 | SD F1 | SL | PD/SL | Firmness |
| --- | --- | --- | --- | --- | --- | --- |
| OS-WB: 800-200 | 3.80 | 1.17 | 0.66 | 2.19 | 1.89 | 5.40 |
| OS-WB: 800-200 | 6.66 | 1.20 | 1.14 | 3.63 | 2.31 | 5.40 |
| OS-WB: 800-200 | 6.66 | 1.20 | 1.14 | 3.63 | 2.31 | 4.30 |
| OS-WB: 800-200 | 3.80 | 1.17 | 0.66 | 2.19 | 1.89 | 4.20 |
| OS-WB: 800-200 | 5.41 | 1.16 | 0.85 | 3.69 | 1.50 | 5.50 |
| OS-WB: 800-200 | 5.41 | 1.16 | 0.85 | 3.69 | 1.50 | 6.20 |
| OA-WB: 800-200 | 5.02 | 1.20 | 0.81 | 2.59 | 2.10 | 7.50 |
| OA-WB: 800-200 | 4.19 | 1.00 | 0.61 | 2.36 | 1.83 | 5.50 |
| OA-WB: 800-200 | 3.99 | 1.05 | 0.58 | 1.89 | 2.31 | 7.30 |
| OA-WB: 800-200 | 5.02 | 1.20 | 0.81 | 2.59 | 2.10 | 6.70 |
| OA-WB: 800-200 | 4.19 | 1.10 | 0.61 | 2.36 | 1.83 | 6.80 |
| OA-WB: 800-200 | 3.99 | 1.05 | 0.58 | 1.89 | 2.31 | 5.90 |
| OLP-WB: 800-200 | 4.79 | 1.33 | 0.96 | 2.72 | 1.82 | 3.66 |
| OLP-WB: 800-200 | 5.11 | 1.21 | 1.07 | 2.21 | 2.47 | 4.40 |
| OLP-WB: 800-200 | 4.79 | 1.33 | 0.96 | 2.72 | 1.82 | 4.50 |
| OLP-WB: 800-200 | 5.11 | 1.21 | 1.07 | 2.21 | 2.47 | 3.80 |
| OLP-WB: 800-200 | 4.79 | 1.33 | 0.96 | 2.72 | 1.82 | 5.10 |
| OLP-WB: 800-200 | 5.11 | 1.21 | 1.07 | 2.21 | 2.47 | 4.90 |
| OA-OLPR-WB: 400-400-200 | 5.95 | 1.30 | 0.95 | 2.65 | 2.30 | 5.00 |
| OA-OLPR-WB: 400-400-200 | 5.11 | 1.21 | 1.07 | 2.21 | 2.47 | 5.00 |
| OA-OLPR-WB: 400-400-200 | 5.02 | 1.20 | 0.81 | 2.59 | 2.10 | 4.80 |
| OA-OLPR-WB: 400-400-200 | 4.56 | 1.29 | 1.04 | 2.79 | 2.08 | 5.80 |
| OA-OLPR-WB: 400-400-200 | 4.79 | 1.33 | 0.96 | 2.72 | 1.82 | 6.10 |
| OA-OLPR-WB: 400-400-200 | 5.00 | 1.20 | 0.80 | 2.50 | 3.13 | 6.20 |
| OS-OA-OLPR-WB: 267-267-267-200 | 4.56 | 1.29 | 1.04 | 2.79 | 2.08 | 7.18 |
| OS-OA-OLPR-WB: 267-267-267-200 | 9.28 | 1.30 | 0.76 | 2.87 | 2.90 | 6.99 |
| OS-OA-OLPR-WB: 267-267-267-200 | 4.56 | 1.29 | 1.04 | 2.79 | 2.08 | 6.45 |
| OS-OA-OLPR-WB: 267-267-267-200 | 9.28 | 1.30 | 0.76 | 2.87 | 2.90 | 7.20 |
| OS-OA-OLPR-WB: 267-267-267-200 | 4.56 | 1.29 | 1.04 | 2.79 | 2.08 | 7.80 |
| OS-OA-OLPR-WB: 267-267-267-200 | 9.28 | 1.30 | 0.76 | 2.87 | 2.90 | 7.40 |
| VIP-WB: 800-200 | 5.67 | 1.60 | 0.80 | 3.20 | 1.77 | 6.50 |
| VIP-WB: 800-200 | 5.31 | 1.49 | 0.80 | 3.10 | 1.71 | 4.60 |
| VIP-WB: 800-200 | 5.48 | 1.50 | 0.70 | 2.70 | 2.03 | 5.20 |
| VIP-WB: 800-200 | 5.52 | 1.50 | 0.80 | 2.50 | 2.21 | 4.30 |
| VIP-WB: 800-200 | 5.05 | 1.28 | 0.70 | 2.10 | 2.40 | 3.30 |
| VIP-WB: 800-200 | 5.21 | 1.39 | 0.70 | 2.20 | 2.37 | 6.70 |
| OA-VIP-WB: 400-400-200 | 4.74 | 1.70 | 0.60 | 2.30 | 2.06 | 5.50 |
| OA-VIP-WB: 400-400-200 | 4.34 | 1.60 | 0.50 | 2.10 | 2.07 | 5.40 |
| OA-VIP-WB: 400-400-200 | 5.54 | 1.80 | 0.70 | 3.10 | 1.79 | 5.60 |
| OA-VIP-WB: 400-400-200 | 4.97 | 1.80 | 0.90 | 2.50 | 1.99 | 5.60 |
| OA-VIP-WB: 400-400-200 | 5.19 | 1.70 | 0.80 | 2.30 | 2.26 | 5.50 |
| OA-VIP-WB: 400-400-200 | 4.89 | 1.70 | 0.60 | 2.40 | 2.04 | 5.40 |
| OS-OA-VIP-WB: 267-267-267-200) | 5.40 | 1.60 | 1.20 | 2.50 | 2.16 | 6.40 |
| OS-OA-VIP-WB: 267-267-267-200) | 3.20 | 0.40 | 0.70 | 1.20 | 2.67 | 5.80 |
| OS-OA-VIP-WB: 267-267-267-200) | 4.80 | 1.40 | 1.10 | 1.70 | 2.82 | 4.30 |
| OS-OA-VIP-WB: 267-267-267-200) | 4.40 | 0.90 | 1.10 | 1.50 | 2.93 | 4.90 |
| OS-OA-VIP-WB: 267-267-267-200) | 4.90 | 1.50 | 1.20 | 2.30 | 2.13 | 6.70 |
| OS-OA-VIP-WB: 267-267-267-200) | 5.10 | 1.60 | 1.30 | 2.10 | 2.43 | 5.00 |
